# An Insufficient Medical Service

**Published:** 1919-08-30

**Authors:** Henry C. Burdett

**Affiliations:** The Lodge, 13 Porchester Square, W. 2.


### THE AFGHAN FRONTIER.

### An Insufficient Medical Service.

Reprinted from the *Times*, August 26, 1919.

Sir,—Since you published my letter on the 12th inst., information has reached me which will strengthen the demand for Government action of the most drastic nature. The treatment of our countrymen in the Medical Service, of which I send particulars, and the consequences of it, show clearly, as I feared, that the grave abuses which prevailed in Mesopotamia must have hardened the hearts of the Indian Government, as Pharaoh's was hardened, with cruel results. They will justly stir the indignation of all classes to insist upon action which will retire the men and change the system which have too long continued to dominate medical affairs in India. I believe, too, that the brief account of Dakka and the conditions and life there will be read with genuine interest and sympathy.

### Indian Government's Mishandling.

The following description of the Indian Government's mishandling of a grave position cannot fail to arouse the just condemnation of the British nation:—

A great many of the British medical officers with temporary commissions are being badly hit by the treatment that the Indian Government is meting out to them. Many were actually on board ship ready to go home, and many more in Bombay awaiting passage, when this war came. They were stopped and taken, hurried up to the frontier and over it. No consideration shown and a pay that no panel doctor in an East-end slum would accept as his only reward. The Indian Government has been ungenerous to them. It always is ungenerous to its Army officers. Some men will undoubtedly be financially ruined. They are frightfully bitter, most of them. Some think they have a remedy in the shape of damages by process of law against the Indian Government. The argument is that they engaged for the European war, and that the Indian Government had no right to take them for a war of its own, and has no right to detain them after the signing of the peace treaty, even if before then it was lawful for it to divert them to the frontier. The legality is doubtful and the hardship in many cases extreme. There is one middle-aged man who bought a practice a few years before the war, increased it, married, and saved money. When the war came he came out on 24s. a day. The practice has dwindled away to nothing; his wife is in financial trouble at home, for now all his savings are spent, his child has died, his wife is ill and has had to undergo a severe operation, and his petitions for release and return are met with an official regret that only urgent cases can be considered.

Few imaginations are equal to figuring a much more urgent case than that of the middle-aged practitioner referred to.

### A Dreadful Place Indeed.

Dakka as it was on July 24 last will bring home to readers of the *Times* what the fighting and conditions our warriors encounter on the North-West Frontier amount to in practice:—

Dakka is a dreadful place. It is on a bare, arid, stony plain, surrounded by bare, arid, shaly hills. Both plain and hills are destitute of trees, of shrubs, of grass. The heat goes up to a great height each day. For weeks it seldom was less than 116 deg. F. in the tents at the hottest of the day. It can be counted as luck if the night is under 100 deg. F. Strong winds often blow and carry vast clouds of dust over the camp, coating everything with a grey, gritty layer. The clouds of dust are often dense as a London fog, and roll up in the wind to the very topmost peaks of the hills. One feels but little energy for work and none for play, even if there were play to be got. By night the camp is sniped several times a week from the north side of the Kabul river. Anything from 20 to a few hundred bullets come whizzing overhead and slapping into the earth. Every one sleeps below the level of the ground, so the casualties are not very many considering the great agglomeration of men and animals that is within the perimeter of the camp.

There are also frequent skirmishes with the enemy a mile or two outside the camp. The road to Jelalabad runs westwards through the hills by a pass called the Khurd Khaiber. Reconnaissances are sent through this frequently to collect such supplies as can be obtained, and to find out what the enemy is doing. This leads to fighting. We find a few thousand of the enemy waiting for us, and there is a fight in which we lose a few men and they usually a good many more. Yesterday we had 22 casualties in such a fight, including one officer and four soldiers killed.

The only amelioration of the discomfort is the daily bathe in the Kabul River. It is a tremendous stream, a quarter of a mile wide at the narrowest, approximately, and very deep and swift. None may swim against the current. The practice is to undress, run up the banks, plunge in, swim down to one's clothes, scramble out again, and repeat the process. Of course, it is only an amusement for practised swimmers.

### The British Government's Driving Force?

Is there no driving force left in the British Government to take swift action and apply the remedy? Surely the holiday fever cannot have swallowed up every man of action amongst them, so that the business of government has come to a dead stop. "Ill can he rule the great that cannot reach the small."

I am, Sir, yours faithfully,

Henry C. Burdett.

The Lodge, 13 Porchester Square, W. 2.

*August* 25.

